# Electrifying Consequences: Posterior Reversible Encephalopathy Syndrome After an Electric Shock Injury

**DOI:** 10.7759/cureus.57821

**Published:** 2024-04-08

**Authors:** Abhinav Ahuja, Sunil Kumar, Aishwarya Gupta, Gautam N Bedi, Maimoona Khan

**Affiliations:** 1 Medicine, Jawaharlal Nehru Medical College, Datta Meghe Institute of Higher Education and Research, Wardha, IND; 2 Obstetrics and Gynaecology, Jawaharlal Nehru Medical College, Datta Meghe Institute of Higher Education and Research, Wardha, IND; 3 General Internal Medicine, Jawaharlal Nehru Medical College, Datta Meghe Institute of Higher Education and Research, Wardha, IND

**Keywords:** neuroimaging, case study, neurological manifestations, posterior reversible encephalopathy syndrome, electric shock injury

## Abstract

Reversible posterior leukoencephalopathy syndrome, often referred to as posterior reversible encephalopathy syndrome (PRES), is a disorder characterized by acute cerebral dysfunction and is seen in conjunction with vasogenic edema on brain imaging. Headaches, visual issues, seizures, abnormal mentation, disturbances in awareness, and focal neurological symptoms are its defining features. In this case report, we present a 40-year-old male patient who developed PRES after experiencing a high-voltage electric shock.

## Introduction

Electrical injuries are a common hazard both in the workplace and at home. Both the immediate and long-term effects of electric shock injury have been linked to a broad range of neurological symptoms. Both instantaneous and delayed onsets are possible with neurological symptoms. These symptoms may develop due to infarction, ischemia, edema, or demyelination. Various anatomical substrates are involved in the neurological manifestations that occur after electrical injury and temporal variability exists concerning electrocution [1].

The clinical-radiologic disease known as posterior reversible encephalopathy syndrome (PRES) can be identified by quickly progressing neurologic symptoms and a distinctive pattern of vasogenic edema on neuroimaging. Headaches, visual abnormalities, seizures, impaired mentation, altered awareness, and localized neurological signs are the hallmarks of PRES [2]. Preeclampsia, eclampsia, the administration of cyclosporine post-organ transplantation, or severe hypertension were the primary settings in which PRES was first reported. However, it can also occur in a wide range of other clinical conditions, including sepsis, infections, shock, chemotherapy, autoimmune illnesses, and hypercalcemia [1]. While PRES has a typical neuroradiological pattern of brain lesion distribution, MRI reveals a symmetrical zone of vasogenic edema of the white matter, largely localized in the areas of posterior circulation [3]. However, in the most severe cases, additional brain areas such as the brainstem, frontal and temporal lobes, basal ganglia, and cerebellum may be affected by the altered signal [4].

## Case presentation

**Figure 1:**
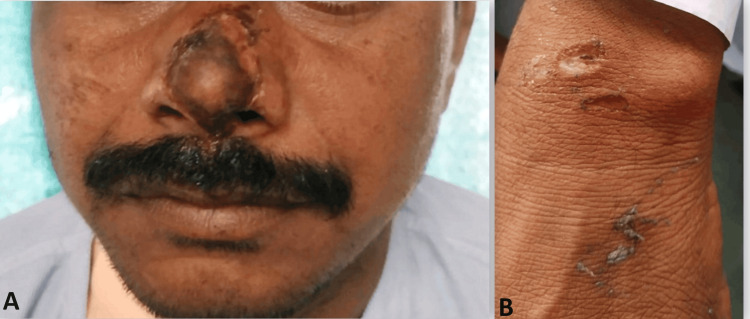
(A, B) Multiple electrical burns.

**Figure 2:**
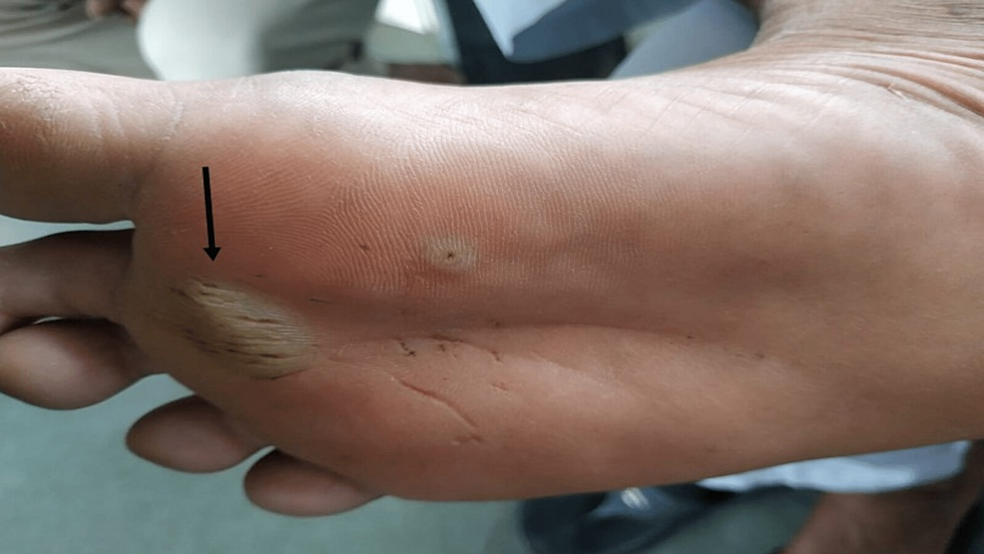
Electrical burn injury seen on the dorsum of the foot.

A 40-year-old male farmer, without any known underlying health conditions, was brought to the urgent care center by his father. He reportedly suffered a high-voltage electric shock on his farm for approximately three to four minutes. Subsequently, he was unconscious for six to seven minutes and experienced several instances of generalized tonic-clonic seizures while en route to the hospital. These seizures were self-terminating in nature. His heart rate was observed at about 150 beats per minute, and his blood pressure was recorded at 180/100 mmHg. As seen in Figure 1 and Figure 2, the examination also revealed the existence of lateral tongue bites as well as multiple electrical burns caused by high-voltage electrical current.

On neurological examination, the patient was drowsy and irritable with altered mentation, bilateral pupils were sluggish but reactive to light, and bilateral plantar flexion and deep tendon reflexes were present. As indicated in Table 1, all standard blood investigations were performed to rule out any imbalance of electrolytes, hypoalbuminemia, protein deficiency, autoimmune etiologies, hypoglycemia, and underlying infections (such as herpes simplex virus (HSV-1 and HSV-2), which may lead to herpes simplex encephalitis).

**Table 1:** Laboratory parameters of the patient with reference ranges.

Investigations	Patient	Reference values
Hemoglobin	13.8 g/dL	13–17 g/dL
Total leukocyte count	7,800/dL	4,000–11,000/dL
Platelet count	345,000/dL	150,000–400,000/dL
Serum creatinine	1.0 mg/dL	0.5–1.2 mg/dL
Albumin	3.8 g/dl	3.5–5.0 g/dL
Aspartate aminotransferase	30 U/L	<50 U/L
Alanine aminotransferase	45 U/L	17–59 U/L
Total bilirubin	0.9 mg/dL	0.2–1.3 mg/dL
Homocysteine	10 mmol	6.6–14.8 mmol
Erythrocyte sedimentation rate	14 mm/hour	0–20 mm/hour
International normalized ratio	0.9	0.8–1.1
Prothrombin time	12.5 seconds	11.9 seconds
Activated partial thromboplastin time	30.5 seconds	29.5 seconds
Creatine kinase	3,200 U/L	55–170 U/L

**Figure 3:**
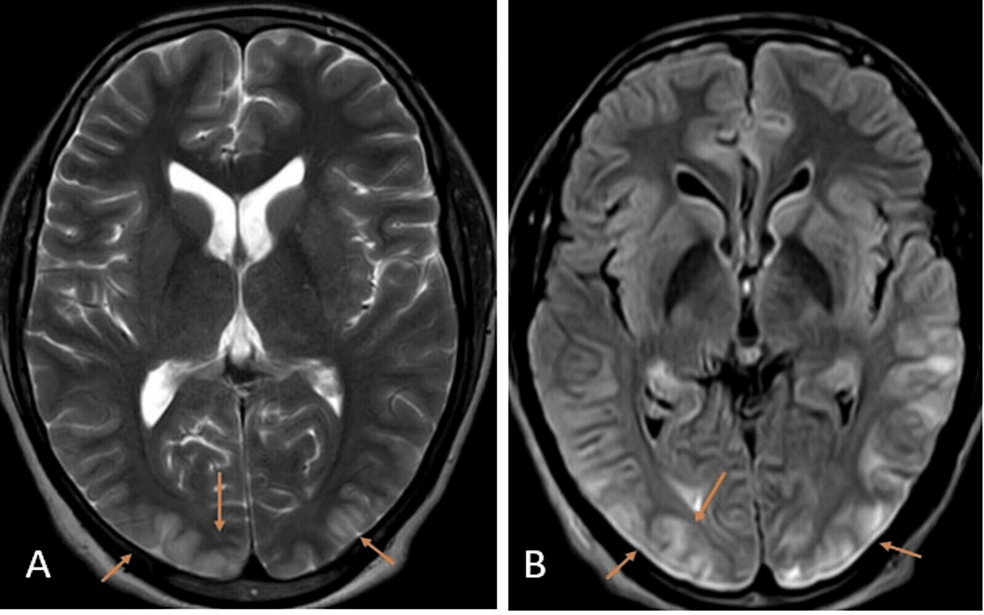
MRI of the brain axial sections T2-weighted imaging (A) and fluid-attenuated inversion recovery sequence (B) showing hyperintensities within the cortex and subcortical region of the bilateral occipital region with associated gyral swelling (orange arrows) suggesting posterior reversible encephalopathy syndrome.

As shown in Figure 3, an MRI of the brain with contrast was performed, which showed vasogenic edematous alterations due to hypoxic-ischemic encephalopathy.

After the results of the brain MRI showed vasogenic edema, the patient received initial treatment in the intensive care unit consisting of intravenous corticosteroids, specifically dexamethasone, at a dose of 4 mg twice daily. Additionally, targeted blood pressure management was implemented using antihypertensive medications such as furosemide injection at 20 mg thrice daily and metoprolol XL tablet at 25 mg twice daily. Antiepileptic therapy was also initiated with levetiracetam injection at 500 mg twice daily to prevent further seizure episodes. The patient regained consciousness after two days of administration of intravenous corticosteroids. With significant clinical improvements and no further episodes of seizures, he was shifted to the inpatient ward. During the recovery phase, the patient complained of persistent headaches. No visual impairment was seen on further examination, and a fundus examination ruled out papilledema.

The patient was discharged after seven days of hospital stay and the neurology opinion suggested the patient was fit for discharge. On a thorough follow-up of the patient, he did not experience any delayed manifestations of PRES and did not report any new complaints.

## Discussion

The various neurological symptoms that can occur after electrical damage are remarkable. These symptoms can range from peripheral neuropathy, epileptic seizures, and central nervous system (CNS) problems (such as myelopathy, encephalopathy, and venous sinus thromboembolism) to mobility abnormalities, migraines, and memory problems. Neuropsychological symptoms may also occur, including sleeplessness, emotional instability, anxiety, electrical fear, difficulty focusing, and post-traumatic stress disorder, among others. To our knowledge, this is one of the few case reports to investigate the link between electrical injuries and CNS disorders [5]. Based on earlier research, we discovered a higher chance of developing CNS disorders in the years after an electrical injury.

Cherington proposed four groups of neurological consequences resulting from electrical trauma: (1) Immediate and brief - symptoms arise during the incident and resolve on their own within hours to days. Examples include sudden loss of consciousness and temporary retrograde amnesia. (2) Immediate and persistent or long-lasting - symptoms emerge during the incident and continue for days for an indefinite period. Common examples include brain hematoma and ongoing infarction. (3) Delayed and progressive - symptoms are not initially present but gradually appear after a variable delay, from days to years post-incident. Conditions falling into this category include progressive demyelinating diseases, movement disorders, hydrocephalus, and thrombosis. (4) Associated or connected - this category encompasses indirect effects, such as head injuries resulting from falls after electrocution [2].

Leukoencephalopathy encompasses various brain white matter disorders, including both hereditary and acquired conditions. As the patient’s electrocution accident predated the availability of MRI, it is challenging to conclusively determine if the observed extensive white matter changes existed beforehand. However, the absence of cognitive or motor dysfunction during childhood, along with the patient’s normal adult functioning, diminishes the likelihood of a pre-existing hereditary leukoencephalopathy or leukodystrophy. Different acquired conditions, such as autoimmune, cardiovascular, infectious, and toxic metabolic causes, were taken into account. Nevertheless, the characteristic white matter changes linked with these conditions, similar to those observed in multiple sclerosis or post-event demyelination, usually manifest as a heterogeneous “patchy” pattern on MRI. Conversely, the leukoencephalopathy observed in the patient presented a homogeneous appearance. In addition, the patient did not exhibit any signs of immunocompromised status. Given a negative neurological examination and inconclusive blood work, most conditions on the initial list of differential diagnoses were effectively ruled out [6].

Although the exact pathophysiology of PRES remains unknown, several theories focus on cerebral dysregulation, which results in changes to the brain’s capacity to dilate or constrict the cerebral blood vessels to maintain consistent cerebral blood flow over a range of blood pressures. The degree of vasoconstriction required to maintain constant cerebral blood flow is greatest at high blood pressures, usually above a systolic pressure of 160 mmHg for most people. Blood flow then starts to climb with rising blood pressure. Elevated hydrostatic pressure may have a role in the blood-brain barrier’s disintegration, which could result in intravascular fluid seeping into the surrounding brain tissue and edema [7].

According to the extra portion theory, an electric field can cause “pores” in the cellular membrane, leading to transient malfunction or cell death. This idea accounts for occasions where electric damage causes temporary symptoms that resolve independently [8]. The pathways described above give us the necessary indication that electrical injury may result in immediate or delayed symptoms such as swelling, myelin loss, reduced blood flow, or tissue death. Some aspects of the pathophysiology remain unclear, including the involvement of different parts of the brain axis, frequency of symptoms, and deeper pathological differences.

Because the posterior circulation lacks the adaptive mechanisms of the anterior circulation to control the degree of extravasation and the breakdown of the blood-brain barrier in the setting of elevated blood pressure, there is a view that the posterior circulation is primarily affected in cases of PRES [9]. However, it was unlikely in this case as the patient was not a known hypertensive. Imaging is crucial in diagnosing PRES, as radiographical evaluation is considered the gold standard. To ensure that neurologic crises, such as cerebral hemorrhage, are ruled out as a cause for an abrupt onset of abnormal mentation, headache, and seizures, a head CT is essential. The preferred imaging method is a brain MRI without intravenous contrast, which can detect hyperintense signals on T2 that indicate vasogenic edema. This edema is commonly observed in the parieto-occipital lobes, which was the case in our patient [7].

If the condition is not treated immediately, PRES complications can arise. As documented in children, complications include focal neurologic impairments from ischemia injury, epilepsy, and potentially fatal diseases, including transforaminal cerebellar herniation [10]. When PRES is detected and treated early, the chances of recovery are high. Symptoms may improve or disappear within a few days to weeks. While there have been a few cases where some visual abnormalities persisted, they typically improve, especially with early treatment of PRES. In most cases, visual symptoms entirely recover. If there is a significant degree of cerebral vasogenic edema, the prognosis may worsen. This is because the increased pressure on the surrounding blood arteries may impair blood flow and cause ischemia. In addition, if the brainstem is involved, the prognosis may deteriorate in certain cases. Currently, there is no clear understanding regarding which individuals are more susceptible to experiencing long-term visual impairments due to PRES. However, it is important to note that delaying therapy for PRES can result in the irreversibility of its symptoms [11]. Therefore, early intervention and prompt treatment are crucial in managing PRES and preventing permanent damage to vision and other neurological functions.

## Conclusions

Timely recognition and treatment of neurological complications such as PRES are crucial after electrical injuries. Prompt intervention with corticosteroids and antiepileptic drugs significantly improved this case. Continued research is essential to refine treatment approaches and improve outcomes for patients with such injuries.
